# Supplementary material for: The petrosal and basicranial morphology of Protoceras celer
Source: PLoS One. 2021 Jul 29;16(7):e0251832. doi: 10.1371/journal.pone.0251832 (PMC8321106; doi:10.1371/journal.pone.0251832)
Supplement: Attachment — Submitted filename: Review The petrosal and basicranial morphology of Protoceras celer.docx [file pone.0251832.s002.docx]

Dear Authors, dear editor,

I have reviewed the article entitled “The petrosal and basicranial morphology *of Protoceras celer*”. Protoceratidae are an interesting and enigmatic family of selenodont artiodactyls. This clade has been considered as part of the basal Tylopoda or the derived Ruminantia. The description here encompass the outer and inner morphology of the basicranial region, including the bony labyrinth and the dorsal surface of the petrosa. Unfortunately, some structures cannot be observed due to preservation and maybe some scanning parameters. This is a precise and complete work. The manuscript is well-written. Maybe it would be easier to follow the discussion if the discussion was in 2 parts: The basicrania in artiodactyls, the protoceratidae basicrania.

I just have some minor comments.

Line 61: Archaeomeryx, which is considered as the basalmost ruminant, do have upper incisors (Janis & Scott 1987, p9)

Line 64: indeed this is a synapomorphy but not an autapomorphy since this is also observed in other clades as the primitive artiodactyls Amphymericidae (Erfurt & Métais 2007).

Line 68-70 do you have a reference?

Line 79-82 What about :

At the turn of the twenty-first century, novel information became available. The endocranial morphology of the basal “leptotraguline” protoceratid *Leptotragulus* was described from physical dissections of the fossil [1] and the derived synthetocerine protoceratid *Syndyoceras* were described was described from computed tomography (CT) scans [25].

Line 83 Joeckel and Stavas [25] and Norris [1].

Norris is the first reference not the 7

Line 111 Could you precise the age of the Poleside member? Maybe with million of year and early Oligocene (?)

Line 126 0.5 mm thickness. Do you mean that the voxel size is 500μm?

Line 133 Do you mean 74.36 μm of voxel size? This may explain the resolution of the semicircular canals. In Mennecart and Costeur 2016 (Tragulidae paper) they used 40 μm.

Line 138-140 which species and which scanning resolution?

Line 145 I think you mean Spoor 29 and not Janis 28.

Line 148 I think calculating the BM for both could be done easily and would be interesting for data on sexual dimorphism.

Figure 1. It would be nice to have it duplicated with the outline of the bones

(see this example from PlosOne <https://journals.plos.org/plosone/article?id=10.1371/journal.pone.0185679>)

Line 177 “on the nasal” not the maxillary? If it is the nasal, then the nasal bones are long (cf line 175). An noted with name of the bones and outlines would help in this case.

Figure 2. It would be nice to have the outline of the bones

Figure 3 is it 70 or 500 μm?

Line 240 It would be nice to have the 3D model to help to follow the description

Figure 4 I would recommend to clean the 3D model deleting (or coloring) the acoustic nerve, the greater petrosal nerve, the petromastoid canal. The cochlea could be “smoothed”/completed using the 3 different views of reconstruction.

Line 294 please delete P. celer

Line 296 What is the pars canicularis?

Line 339 “The cochlear aqueduct housed the perilymphatic duct in life.” This is not description and do you have a reference?

Line 372 Janis proposed 40 Kg. Could you please calculate the weight for the second specimen to have an idea of the sexual dimorphism?

Line 374-377

When applied to the appropriate agility predictive equation (see Materials and Methods), we recover two agility scores. Using the full skull length body mass, we predict an agility score of 2.97. Using the basicranial body mass, we predict an agility score of 3.057.

Is it what you mean?

The agility score obtained from the body mass calculated from the skull length is of 2.97 while it is of 3.057 using the body mass based on the basicranial.

Line 390 the Protoceratidae *Paratoceras*

Line 417-418 Joeckel and Stavas [25] observed that *Syndyoceras* has a thin bony process extending from the basioccipital to the bulla.

Line 419-421 Scott [10] reported that the bulla and basioccipital of *Protoceras* are too closely appressed for the petrosal to be visible through the gap.

Line 449 Could you specify the family of all these artiodactyls.

In this article Cainotheriidae and Anaplotheriidae are thought to be sister taxa of the ruminant

<https://www.researchgate.net/publication/346096394_A_new_Cainotherioidea_Mammalia_Artiodactyla_from_Palembert_Quercy_SW_France_Phylogenetic_relationships_and_evolutionary_history_of_the_dental_pattern_of_Cainotheriidae>

Line 478 which Camelid?

The Homacodontidae/basal artiodactyl *Bunomeryx*

What about the Merycoidodontidae (Tylopoda?) ? There are closer chronologically with the *Protoceras* than living ruminants and maybe more closely related. There is a *Merycoidodon* described in O’Leary (Fig 74)

Line 495 the Eocene camelid *Poebro* and the extant *Lama*

Line 506 Indeed + there is a lack of quantification of the structure so the definition of deep or shallow may vary.

Line 512 Is there a mastoid fossa in *Merycoidodon*?

Line 538 likely that this

Line 588 the basicapsular groove is variable within the ruminants

Line 589 what means an intermediate protoceratine?

Line 616-917 In ruminants the basicapsular groove is variable and seems to be phylogenetically informative. It has been used in cladistics analyses published in PlosOne (Aiglstorfer et al. 2017, Mennecart et al. 2021)

<https://journals.plos.org/plosone/article?id=10.1371/journal.pone.0185679>

<https://journals.plos.org/plosone/article?id=10.1371/journal.pone.0244661>

Line 645-647 Please consider that the external morphology of the petrosal suffers of a strong ontogenetic shape allometry. The specimen lacks a mastoid region and is very rounded as observed in calf (Costeur et al. 2017). Moreover, since the petrosal is isolated, it can be a juvenile.

<https://onlinelibrary.wiley.com/doi/full/10.1111/joa.12549>

However the subarcuate fossa is extremely deep and I fully agree with your conclusion.

Line 657 there should not be references in the conclusion

Line 719 *Archaeomeryx* should be in italic

The doi website is strange

Line 740 The doi website is strange

Best regards,

Bastien Mennecart
