# Supplementary material for: The petrosal and basicranial morphology of Protoceras celer
Source: PLoS One. 2021 Jul 29;16(7):e0251832. doi: 10.1371/journal.pone.0251832 (PMC8321106; doi:10.1371/journal.pone.0251832)
Supplement: Attachment — Submitted filename: Response_to_Reviewers.docx [file pone.0251832.s003.docx]

Editor

1. We have added the corresponding author’s email address to the title page, and we have changed our Level 1 and Level 2 headings to match journal requirements. We did not have any Level 3 headings to modify. To our knowledge, our figure captions and equations are formatted correctly. Equations were inserted using the Insert 🡪 Equation function in Word. We do not have any inline equations in our manuscript. All inline symbols were inserted using the Insert🡪Symbol function. We have done our best to name the files according to the PLOS ONE style requirements.

2. The conference abstract was not peer-reviewed and/or formally published and should not violate the rules of dual publication.

3. We apologize for confusion about data accessibility. We were under the impression that we needed to provide the information in the Data Accessibility section but not the manuscript. We have added the DOIs and relevant instructions to the body of the manuscript. We are unable to make some data publicly accessible because the data are owned by the American Museum of Natural History (AMNH). We have provided instructions in our manuscript on whom to contact at the AMNH to request permission to download the data. Restrictions are outlined by the AMNH here: <https://www.amnh.org/research/paleontology/3d-scanning>, under “Copyright, Licensing, Use and Distribution of 3-D data generated from specimens in the AMNH Division of Paleontology collections”. We see no reason why the AMNH would deny a download request, and the specimens are accessible for examination and re-scanning by interested parties, but the final decision lies with the museum. All data not owned by the AMNH are publicly accessible on MorphoSource.

4. We do not have any supporting information files for this manuscript.

Note: We have updated the phrasing in our acknowledgements to reflect the preferences of the named parties.

Reviewer 1

Line 99: We have changed “explanations” to “scenarios”.

Line 101-103: We recognize that including conclusions is a spoiler. We included this statement because PLOS asks that we conclude our introduction “with a brief statement of the overall aim of the work and a comment about whether that aim was achieved”. We have attempted to revise our statement to reduce spoilers while still complying with the journal.

Line 129: The resolution was in pixels. We have added voxel sizes and removed the resolutions because they are now redundant.

Line 167: We have clarified that we are discussing the cranium.

Line 228-230: We have updated our figures to better-show the morphology.

Line 384: We have added some text to express the relevance of cainotheriids. The text does not fit particularly well in this line, so we added it to the next mention of cainotheriids.

Line 496-497: Good point about the character polarities. We have modified the text and no longer suggest that a deep subarcuate fossa is unusual.

Line 511-512: We agree that the mastoid fossa could easily be a symplesiomorphy. So far, most descriptions of basal artiodactyl petrosals have not made mention of a mastoid fossa, but we would be interested in exploring this further. It is possible that a mastoid fossa was present and was simply not described. Given that we currently have limited data, we are choosing not to make such an assumption in our manuscript and are rather listing the taxa that are known to have a mastoid fossa.

Organization: We have reorganized the descriptions as per the reviewer’s suggestion. We thank the reviewer for providing us with an outline for the organization. We have chosen to keep the body mass and agility score estimates separate because, while they are inferences based on the data in our description, they are not actually part of the description.

Data accessibility: We were unsure of where to put the DOIs, so we reported them in the submission form but not the manuscript. We are in contact with the editor to discuss data availability. We apologize for not including the relevant information in the manuscript.

Figure 1: We have modified this figure by adding outlines to the specimens. We think that this enhances the visibility of the structure we discuss in the text, including the auditory bulla.

Figure 2: We have modified this figure by adding outlines to the CT renderings. As with Figure 1, we think that this enhances the visibility of structures which were previously unclear.

Reviewer 2

Line 61: We have clarified the sentence to say *most* ruminants.

Line 64: We fully agree that a cubonavicular is not a ruminant autapomorphy and we were not intending to suggest differently. The purpose of our statement was to indicate that protoceratids lack a cubonavicular and, as such, lack an important ruminant synapomorphy. We did not refer to amphimerycids as we did not think they were relevant to the discussion at hand. We have left the text as-is because we are concerned that mention of amphimerycids would detract from the comparison of ruminants and protoceratids, but we welcome suggestions on rephrasing if the reviewer feels strongly on this point.

Line 68-70: We have added several references that discuss plesiomorphic and convergent morphology in artiodactyls.

Line 79-82: We have changed the wording to what the reviewer suggested. We thank them for helping us with phrasing.

Line 83: We have added the numbered references. The numbers have changed since the first submission because of manuscript reorganization.

Line 111: We have added dates for the Poleslide member.

Line 126: We have changed the wording to indicate a voxel size of 500 microns.

Line 133: We have changed the wording to indicate a voxel size of 63.4765 x 63.765 x 0.07436 µm. We do not think that the relatively large voxel size would have much of an impact on the semicircular canal resolution as the skull of *Protoceras* is much larger than the skulls of the tragulids, but we will keep this in mind for future scanning endeavors.

Line 138-140: We have added the taxon names and scanning parameters. The comparative data are uploaded to MorphoSource with public access.

Line 145: The reviewer is correct. We did mean Spoor et al. (2007) and we have fixed the citation.

148: We have added body mass calculations for AMNH-VP 1229 and have included a body mass comparison in our discussion.

Figure 1: We have modified this figure by adding outlines to the specimens. We thank the reviewer for the suggestion and for pointing us to example images.

Line 177: We did mean the maxillaries, not the nasals, and we have amended our description.

Figure 2: We have modified this figure by adding outlines to the CT renderings.

Figure 3: All figures of AMNH 53323 are from the high-resolution scan.

Line 240: We agree that it would be nice to provide the 3D models, but all scan data, including the 3D renderings, belong to the AMNH and therefor cannot be provided publicly. We have done our best to illustrate our descriptions using our figures.

Figure 4: We have added colours to the 3D model to help distinguish features. We were unable to enhance the quality of the cochlea as it is already smoothed and reconstructed based on all three views; unfortunately, the bony labyrinth of the specimen is simply not that well preserved.

Line 294: *P. celer* has been deleted.

Line 296: The “pars canicularis” is an unfortunate misspelling of “pars canalicularis”. We have fixed the mistake.

Line 339: We have removed this sentence as we agree it is not part of the description.

Line 372: The 40 kg proposed by Janis was based on the lower molar row length, which is not something we could calculate as we only have skulls. Our posterior skull length calculation produced a body mass of 22.0 kg and our total skull length calculation produced a body mass of 27.3 kg. Based on the same metrics, Janis estimated a body mass of 29.4 (SD 28.6) and 37.0 (SD 10.0), respectively. This is why we said that our estimates were similar to those from Janis. We have elaborated on our discussion of body mass, including a comparison to the second specimen and a discussion of potential sources of error. We thank the reviewer for encouraging us to explore the matter further.

Line 374-377: The reviewer was correct in interpreting our meaning. We have amended our language to make ourselves clearer.

Line 390: We have clarified that *Paratoceras* is a protoceratid.

Line 417-418: We have added the numeric citation in the correct location.

Line 419-421: We have added the numeric citation in the correct location.

Line 449: We have added the family names to the taxa.

We included anoplotheriids and cainotherioids under the classification of “purported tylopods” because they have frequently been referred to Tylopoda in the literature, even if this position is disputed (e.g., we consider protoceratids to also be purported tylopods). We used the term “purported” to indicate that not all listed taxa necessarily belong to the Tylopoda. We have kept the sentence as-is for the moment as we hope this clarification will suffice, but we would be happy to rephrase if the reviewer has other suggestions.

Line 478: We are referring to both extinct and extant camelids. We have clarified this in the sentence, and we have clarified that *Bunomeryx* is a homacodontid. Also, thank you for noting that we omitted *Merycoidodon* from this list. The taxon has now been added.

Line 495: We have now indicated that *Poebrotherium* is extinct and *Lama* is extant.

Line 506: Absolutely! Some quantification would certainly be welcome. We have added a sentence expressing this problem at the end of the paragraph.

Line 512: *Merycoidodon* has a shallow depression which may be a reduced mastoid fossa, and *Ticholeptus* might have a deeper mastoid fossa. We have added a comment about oreodonts to our discussion of the mastoid fossa.

Line 538: We have fixed the duplicate word.

Line 588: This figure is a modified version of figures published by Norris (1999) and Theodor (2010). Thanks to the reviewer’s comments, we have further modified the figure to show that ruminants have a basicapsular groove. We have also changed the figure heading to indicate that the basicapsular groove is being used to infer the position of the inferior petrosal sinus.

Line 589: By intermediate, we mean a protoceratid that is neither basal nor highly derived. We have changed the wording to say “relatively underived” rather than “intermediate”. Hopefully this clarifies our meaning for the figure. We have retained the word “intermediate” in other portions of the manuscript where contextual cues make our meaning more clear.

Line 616-617: We used extant ruminants to illustrate our point that the presence of a basicapsular groove cannot solely be used to infer the location of the inferior petrosal sinus. We did not cite Aiglstorfer et al., (2017) and Mennecart et al. (2021) because, while excellent descriptions, these papers focused on extinct ruminants and did not infer the location of the inferior petrosal sinus. We agree that the position of the basicapsular groove may be phylogenetically important and should be explored further, but this was not the intended focus of the example.

Line 645-647: We have added additional discussion about the identity of AMNH-VP 645 following the reviewer’s advice. We thank the reviewer for these helpful insights.

Line 657: We have removed the reference.

Line 719: *Archaeomeryx* became italicized when we refreshed out citations. The DOI was for the wrong article and we have removed it.

Line 740: The DOI appears to be correct, and we have confirmed that it links to the right article. We do not know why it previously looked odd.

Reviewer 3

We thank the reviewer for directing our attention back to the figures in O’Leary (2010). We agree that, aside from the subarcuate fossa, the endocranial surfaces of AMNH-VP 645 and our described *Protoceras* petrosal are similar. We have re-checked our scans and have found no evidence of infilling, and we do not think that this is a likely scenario given that the path of petromastoid canal through the subarcuate fossa is clearly defined in our scan. We cannot discount potential polymorphism in the taxon and we have added such a suggestion to our manuscript, although we do note that subarcuate fossa polymorphism has not been described in other artiodactyl taxa.

In addition to the discrepancy in subarcuate fossa size, we have noted a couple of additional differences between specimens. AMNH-VP 645 has a distinct notch on the tympanic face dorsal to the epitympanic wing. Our specimen lacks this notch. There is also a large difference in the shape of the mastoid region; AMNH-VP 645 lacks a distinct mastoid process whereas our described specimen has a large process. We have added these comments to the paper. One reviewer has suggested that these differences could be ontogenetic in nature—AMNH-VP 645 may be from a juvenile—but observed that the deep subarcuate fossa did not support this hypothesis. We have also added this aspect of discussion to our paper. We have removed our suggestion that AMNH-VP 645 may be from a basal camelid as we agree that this speculation is unnecessary and requires more data to be supported.

1. Line 86: We agree our phrasing was awkward, and we have changed it to an active voice.
2. Line 90: Thank you for drawing our attention to Fig. 41. We have removed our statement about the endocranial ridge.
3. We have added additional discussion of the body size estimates, including estimates for the female skull and comments on why some of the size estimates may be off.
4. We acknowledge that some of our descriptions are redundant with previous descriptions of *Protoceras celer*. However, we have chosen to retain our descriptions given that AMNH-VP 53523 and AMNH-VP 1229 have not previously been described in detail.

We have added a paragraph explaining why these two specimens have been referred to *Protoceras*. We thank the reviewer for the suggestion.

5. Line 276: This should say lateral process. We checked our manuscript and it appears correct in our version, which is why the line does not show any tracked changes. We are not sure why it was incorrect on the reviewer’s copy, but we apologize for the error.
6. Line 279: We have relabeled the figure with “posteromedial” flange.
7. Line 318: The mastoid plate is a structure present in extant suines. We have added a reference (O’Leary, 2010) for a definition.
8. Line 320: We have added a reference (Orliac and O’Leary, 2014).
9. Line 359: We have corrected the spelling error.
10. Line 549-551: We are a bit confused as to what is being asked here. The reviewer says that a structure has not been described in *Dissacus*, and then goes on to suggest we might be confusing the structure with the petromastoid canal. We are confused because the paragraph in question is indeed discussing the petromastoid canal and, in our statement about *Dissacus*, we only refer to the petromastoid canal. We are not sure what structure the reviewer meant, and we would greatly appreciate clarification so that we can correct any errors we have made.
11. Fig. 5: We have labeled the region of the endocranial ridge. The ridge itself is best viewed in cross-section, as shown in Figure 6, but we hope that indicating the correct region is helpful.
